# Special collection on “Stroke Management and Therapy” accompanying the 99th congress of the german society of neurology

**DOI:** 10.1186/s42466-026-00548-7

**Published:** 2026-11-02

**Authors:** Götz Thomalla, Wolf-Rüdiger Schäbitz

**Affiliations:** 1 Universitätsklinik für Neurologie, Universitätsklinikum Hamburg-Eppendorf, Hamburg, Germany; 2 Universitätsklinik für Neurologie, Evangelisches Klinikum Bethel, Universität Bielefeld, Bielefeld, Germany

Worldwide, stroke is one of the leading causes of death and, given the rising absolute number of cases, represents one of the greatest global health challenges. Although the prognosis for stroke in Germany has improved thanks to modern treatment options, it remains the most common cause of permanent disability in adulthood.

Thanks to modern imaging techniques, it is now possible to perform thrombolysis treatment within a significantly wider time window, even for so-called “wake-up strokes.” Mechanical thrombectomy has undoubtedly revolutionized stroke treatment—both for large vessel occlusions in the anterior circulation and for basilar artery occlusion. Care on a stroke unit is standard in Germany and has led to a significant improvement in prognosis. Important progress has also been made in the field of secondary prevention: innovative treatment approaches have already found their way into clinical care or are currently being investigated in clinical trials. The motto of the 99th Congress of the German Society of Neurology (DGN) and the Neurowoche, “Vascular Neurology – Innovation, Precision, Prevention,” therefore comes at exactly the right time.

Great advances in research and clinical care are no longer the result of isolated national efforts, but predominantly stem from international collaborations. Therefore, we are very pleased to welcome our important international partners from the European Academy of Neurology (EAN), the American Academy of Neurology (AAN), the World Federation of Neurology (WFN), and the European Stroke Organisation (ESO) to the Neurowoche. The Neurowoche also reprensents an interdisciplinary event involving our partners from neighbouring disiciplines, including Neuroradiolopgy, Neurosurgery, Cardiology, Neuropathology and Neuroanatomy, and others. Together, we want to discuss the global interdisciplinary challenges and current developments in vascular neurology.

New evidence from clinical trials forms the basis for changes in guidelines and clinical practice. This applies in particular to vascular neurology. Many groundbreaking studies on stroke diagnosis, treatment and prevention in the past and the present originate from Germany. This ongoing activity is reflected by the newly established Stroke Specialist Network within the University Medicine (NUM-STROKE), which is intended to make the planning and implementation of clinical stroke trials even more effective in the future and possibly to generate new evidence which may change clinical practice.

In a special collection on Stroke Management and Research published parallel to the conference in DGN´s premier journal, *Neurological Research and Practice*, the study master protocol of the Stroke-Core, the prospective cohort of the Speciality Network Stroke (SN Stroke) within the German Network University Medicine (NUM), is presented [1]. This protocol is intended to create and represent a large national stroke cohort to gather novel information on stroke management and hopefully derive new momentum for developing future diagnosis and therapy. Other content includes an article on the reality of stroke management in Germany, [2] the efficacy of mechanical thrombectomy for stroke treatment, [3] the diagnosis and management of patients with small vessel disease and microbleeds, [4] as well as three state of the art review articles on the implications of Factor XI and factor XII inhibitors for stroke treatment and prevention, diagnosis and treatment of atrial fibrillation and pneumonia [5–7].

The articles in this special collection are complemented by dedicated sessions in the scientific programme of the DGN Congress and Neurowoche. In the session entitled “New Concepts for Clinical Trials – Stroke Trials ‘Made in Germany’” the concept of the Network of University Medicine (NUM) study network and its Specialty Network Stroke will be presented. Further scientific sessions, organised jointly with international experts and the German Society of Neuroradiology, will focus on the current state of mechanical thrombectomy as well as emerging perspectives in neurointervention and stroke imaging. A joint session with the German Society of Cardiology will provide an update on topics at the interface between the heart and the brain. Finally, the Presidential Symposium will address the systemic dimensions of stroke, including its immunological sequelae and the role of cerebral small vessel disease.

We hope that you enjoy this special collection and that it provides both an overview of current developments and inspiration for future research and clinical practice. We warmly invite our readers to join us at the DGN Congress and Neurowoche, where the topics highlighted in this collection will be presented and discussed in greater depth in the corresponding scientific sessions.
